# Construction of an Index System of the Biosafety Incident Response Capability for Nursing Staff: A Delphi Study

**DOI:** 10.1002/nop2.70118

**Published:** 2024-12-30

**Authors:** Chao Wu, Mengyi Hu, Xinyan Zhang, Mimi Fu, Lu Li, Qiang Xu, Xiaolan Guo, Hongjuan Lang

**Affiliations:** ^1^ Department of Nursing Air Force Medical University Xi'an Shaanxi China; ^2^ Department of Medical Engineering Army 75th Group Army Hospital Dali Yunnan China; ^3^ Department of Pharmacy Sanya Rehabilitation Center Sanya Hainan China; ^4^ Department of Anesthesia Intensive Care Unit, the Second Affiliated Hospital Air Force Military Medical University Xi'an Shaanxi China; ^5^ Lintong Rehabilitation Convalescent Center of Joint Logistics Support Force Lintong Shaanxi China; ^6^ Department of Cardiovascular Medicine Air Force Medical University Xi'an Shaanxi China

**Keywords:** biosafety incident, coping competence, Delphi method, index system, nursing staff

## Abstract

**Aim:**

This study was to establish a scientific and sensitive index system of the biosafety incident response competence for clinical nursing staff to provide a reference for the evaluation of nurses' biosafety incident response competence.

**Design:**

A modified recommendation for the conducting and reporting of Delphi studies was used to guide this study.

**Methods:**

According to the literature review, preliminary biosafety incident response competence indicators for nursing staff were established, and an expert survey questionnaire was designed. The evaluation system for clinical nurses' biosafety incident response competence indicators was determined using the Delphi method.

**Results:**

After two‐round Delphi survey, 28 nursing specialists from six provinces and cities around China, with expertise in three prevention research, epidemiology, military health service and biosafety incident rescue, established the index system of the biosafety incident response competence for nursing staff from April to May 2023. The final index system of the biosafety incident response competence was composed of four primary indicators, namely, biosafety incident preparedness, monitoring competence, protection ability and nursing disposal abilities; 10 secondary indicators and 49 tertiary indicators. The effective recovery rates of the two rounds of expert survey questionnaires were 93.33% and 100%, respectively. The authority coefficient, judgement coefficient and familiarity degree of Delphi experts were 0.877, 0.911 and 0.843, respectively. The Kendall's harmony coefficients of the two rounds of expert consultation were 0.301–0.384 and 0.401–0.424, respectively, with statistical significance (*p* < 0.05).

**Conclusion:**

The index system of the biosafety incident response competence for nursing staff is scientific and reliable. The authors have provided a more reliable and comprehensive basis for evaluating nurses' biosafety incident response competence in the future.

**Conclusion and Implications for Nursing:**

It is helpful for nursing staff to perform better in biosafety incident by clarifying the specific items of biosafety response competence. Nursing staff can use the index system as the evaluation tool and training references to enhance their biosafety incident response competence.

**Patient or Public Contribution:**

No patient or public contribution.

## 1 Introduction

With the development of economy, trade and transportation, globalisation, industrialisation and urbanisation have led to the acceleration of the spread of pathogenic microorganisms and the increase in transmission routes (Liu et al. 2019). What is more, the melting of glaciers brought by global warming results in the release of pathogenic microbes from ancient times in harsh habitats (Yun et al. 2022). All of these factors have increased the time it takes for biological threats to develop and hide, as well as the time and space they can infect people (Li et al. 2018). They have also led to an unprecedented rise in biosecurity and bioterrorism threats. Traditional biosecurity problems suchas major infectious diseases, biological weapons, laboratory biosecurity and biological invasion are intertwined with non‐traditional biosecurity problems like the dual use of biotechnology and cyber biosecurity in the context of the new round of scientific and technological changes as well as the transformation of the global political and economic order, leading to increasingly serious national security issues (D. Zhou et al. 2019). It significantly affects both national and global patterns of development (Das et al. 2021; Naik et al. 2021).

The ability of nursing staff to respond to major emerging infectious diseases, pathogen microorganism laboratory leakage, microbial drug resistance, biological weapons threat, bioterrorism attack and other biosecurity incidents, such as emergency preparation, monitoring and early warning, protection and sensitivity control, nursing disposal, and so on, in order to stop the spread of biosecurity incidents, is known as the “biosafety incident response competence for nursing staff”. The effectiveness of the overall medical system's response time will be strongly impacted by the nursing staff's biosafety response competence and response speed because they are the frontline medical and health security forces and the first line of biosafety treatment (Curtis et al. 2020; Frankfurter 2019). In this way, every nurse must process this competence to respond to biosafety threats effectively (Gilbert and Kerridge 2022).

The biosafety incident response ability of nursing staff is systematic disposal ability. In the wake of the September 11 terrorist attacks, the Centers for Disease Control and Prevention (CDC) identified the abilities that all public health personnel need to have when responding to terrorist attacks and public health emergencies, which serves as a benchmark for their future ability to handle such attacks and emergency rescue (K. Gebbie and Merrill 2002). Through the Delphi study, Barbara J. Polivka et al. (2008) highlighted the competence framework that local nursing managers and public health nurses need to have while handling medical emergencies. This framework primarily addresses the competence of planning, reaction and recovery. Gebbie et al. (2008) clarified the legal knowledge and ability that medical personnel should possess when responding to public health emergencies. Emerging infectious diseases and biological weapons are currently posing a severe threat to all of humanity due to climate change and global instability (Volk and Gering 2021). Every member of the clinical nursing staff now needs to be proficient in biosafety response competence; it is no longer just a skill that public health nurses, nursing supervisors or military nurses need to master. Therefore, in order to respond to potential biosafety threats at any time, this study will clarify the biosecurity skills that all clinical nurses need to possess.

However, a significant limitation of most existing general healthcare competence frameworks is their lack of specific focus on biosafety incidents within the nursing context. There is a dearth of studies that evaluate nursing staff's competence in dealing with biosafety incidents and emergencies, such as handling hazardous biological materials, implementing specific isolation procedures, responding to bioterrorism attacks and managing potential infections among patients. Additionally, very few index systems emphasise the integration of different dimensions related to biosafety incident response, including knowledge, skills and attitudes. In particular, there is a lack of focus on the psychological resilience and decision‐making abilities of nurses under high‐pressure biosafety situations. To address these gaps, we developed an index system for nursing staff's competence to respond to biosafety incidents through literature review and group discussion. This index system was then modified and improved through Delphi study with experts from the fields of prevention research, epidemiology and military health service, and clinical medical and nursing experts who had participated in biosafety rescue. Finally, we have created an index system for nursing staff's competence to respond to biosafety incidents in order to counter the current threat.

## 2 Methods

### 2.1 Establishment of Research Group

The research group was set up before the research, including one professor, two doctoral students in nursing and two master's students in nursing. The members of the research team were mainly responsible for the literature review, the questionnaire development and consultation with experts.

### 2.2 Literature Retrieval and Review

The research group searched a large number of relevant studies through searches of PubMed, Web of Science, Embase database, WEip.cn, CNQI and Wanfang database (from inception to March 2023). The keywords included nursing staff, biosafety incident, coping competence, Delphi method and index system. The research team sorted and screened the relevant studies, discussed and studied them together. According to the literature review, preliminary biosafety incident response competence indicators for nursing staff were established.

### 2.3 Delphi Survey

After preliminary biosafety incident response competence indicators for nursing staff were established, a Delphi survey was used to compile and synthesise the expert feedback. The Delphi technique reporting criteria involve panel selection, round design with feedback, data analysis for consensus measurement and clear reporting of the process and results including details of each step.

Through focused feedback, we got the outcomes until all expert viewpoints were in agreement with the final index system (McMillan, King, and Tully 2016). In the process of study, experts did not know each other, which could ensure the independence of expert opinions and the reliability of research results (McPherson, Reese, and Wendler 2018; Smart et al. 2010).

#### 2.3.1 Experts Selection

It was commonly accepted that the number of experts consulted was generally between 15 and 30 (Hasson, Keeney, and McKenna 2000). The more experts were involved, the more reliable the outcomes were. We would select experts in research fields related to this subject to conduct correspondence consultation on the index system.

To ensure the representativeness of the expert panel, we first established clear and stringent inclusion criteria. Experts were sourced from a diverse range of relevant fields. Specifically, we included those with rich clinical nursing experience in biosafety‐related departments, who had been actively involved in day‐to‐day patient care within the context of biosafety, handling complex situations and applying their practical knowledge. Additionally, professionals engaged in biosafety research and management in the medical environment, who were well‐versed in the latest scientific advancements, regulatory requirements and risk mitigation strategies, were incorporated. Moreover, educators who had trained nursing staff in biosafety knowledge and skills, bringing in pedagogical insights and understanding of knowledge dissemination in this critical area, were also part of our selection pool. This multi‐faceted approach aimed to cover different perspectives and experiences related to nursing and biosafety incident response comprehensively.

During the selection process, we deliberately considered geographical distribution to avoid over‐concentration of experts from a particular region. We reached out to a wide array of hospitals and research institutions across different geographical locations. This was done to incorporate diverse practices and insights, ensuring that the panel was not skewed towards a specific local context or set of practices.

Furthermore, we employed a snowball sampling technique. After an initial group of experts was identified based on the established criteria through extensive literature review, professional network referrals and institutional recommendations, they were contacted and asked to recommend other potential experts. We provided them with detailed guidelines on the expertise and experience we were seeking, ensuring that the recommended individuals met our predefined requirements. This method helped us to identify experts who might not have been easily reached through traditional means, such as published research databases or professional associations alone, and further enhanced the comprehensiveness of the panel.

The inclusion criteria of consultation experts were as follows: (a) experts in the three prevention research fields, epidemiologists, experts from the military health service and clinical medical and nursing experts who had taken part in biosafety rescue; (b) their working years were no less than 10 years; (c) they should obtain the intermedium or advanced level certificate and (d) they should provide informed consent and voluntarily participate in the investigation.

#### 2.3.2 Ethical Considerations

In strict adherence to the Declaration of Helsinki (Dal‐Ré 2023), we placed paramount importance on the ethical treatment of participants throughout the entire process. First, regarding voluntary participation, we provided each potential expert with a detailed description of the purpose, scope and expected time commitment of the consultation project. This was accompanied by a clear explanation of how their input would contribute to the development and improvement of the index system related to biosafety in the nursing field. We emphasised that participation was entirely optional and that they could withdraw at any stage without any negative consequences. To formalise this, we obtained written informed consent from each expert, which clearly stated their understanding of the project and their willingness to participate. The consent form was designed in plain language, easily understandable by all participants, and was reviewed by an independent ethics committee to ensure its adequacy and compliance with ethical standards.

Confidentiality was another crucial aspect of our ethical approach. We implemented a strict data protection protocol where all personal information, professional experience details, research focus and any other data provided by the experts were stored securely in encrypted databases. Any communication related to the consultation, whether in written or verbal form, was anonymised whenever possible to protect the identity of the participants. For example, when presenting aggregated findings or quoting expert opinions during the Delphi rounds, we used codes or generic descriptors instead of revealing personal identities.

Finally, we took every measure to ensure no harm was caused to the participants. We carefully designed the consultation process to minimise any potential burden on their professional or personal lives. The correspondence consultation was scheduled in a flexible manner, allowing experts to respond at their convenience within reasonable time frames. In case any expert expressed concerns or discomfort during the process, we immediately addressed their issues, either by adjusting the consultation requirements or providing additional support and clarification. Overall, our commitment to ethical considerations was an integral part of the expert selection and consultation process, safeguarding the rights and well‐being of all involved participants while striving for the highest quality research outcomes.

#### 2.3.3 Basic Information of the Experts

In order to make the selected experts more representative, the research team selected 28 experts from six provinces and cities of Beijing, Chongqing, Shanghai, Shaanxi, Shanxi and Zhejiang, to participate in the survey of the index system of the biosafety incident response competence for nursing staff. Their age ranged from 37 to 60 years, with an average age of 48.53 (SD 5.88) years. Their working years varied from 13 to 36 years, with an average of 25.04 (SD 5.85) years. Other basic information is shown in Table 1.

**TABLE 1:** Demographic information of experts.

Project	Group	Frequency (*N*)	Proportion (%)
Age (years)	< 40	2	7.14
	40 ~ 50	15	53.57
	> 50	11	39.29
Working years (years)	< 20	3	10.71
	20 ~ 30	18	64.29
	> 30	7	25.00
Research field	Three prevention research	5	17.86
	Epidemiology	8	28.57
	Military health service	7	25.00
	Clinical medical and nursing	8	28.57
Title	Senior—I Professional title	11	39.29
	Senior—II Professional title	17	56.67
Highest degree	Doctorate	12	42.86
	Master's	11	39.29
	Undergraduate	5	17.86
Region	Beijing	4	14.29
	Shanghai	3	10.71
	Chongqing	5	17.86
	Shaanxi	11	39.29
	Shanxi	3	10.71
	Zhejiang	2	7.14

#### 2.3.4 Consultation Questionnaire

The questionnaire was compiled by the research group and was composed of three parts: (a) general information of the experts, (b) the index system of the biosafety incident response competence for nursing staff consultation form and (c) expert familiarity with the content of the survey and index judgement (as shown in Supplementary file).

#### 2.3.5 Delphi Expert Consultation

The first round of Delphi survey was conducted in April 2023 by sending questionnaires to the selected experts by email. Experts were given 2 weeks to fill in the questionnaires to ensure the quality of their opinion. We redesigned the questionnaire in light of expert opinion after the first‐round consultation.

On the basis of the first round, the second‐round consultation was launched in May 2023. Giving the selected experts 2 weeks to complete the questionnaires, we collected and analysed their opinions and scores to establish the index system of the biosafety incident response competence for nursing staff.

### 2.4 Statistical Analysis

SPSS 26.0 statistical software was applied in the process of data analysis. Data measurement and data calculation were expressed in the form of mean ± standard deviation and frequency, and percentage, respectively. The enthusiasm of the experts was also expressed in the form of questionnaire recovery rate. The expert authority coefficient was the average value of expert familiarity with the indicators (Cr) and judgement criteria for the indictors. The coordination degree of expert opinion was presented by Kendall harmony coefficient.

## 3 Results

### 3.1 The Effective Recovery Rates of Experts

In the first round of our study, we meticulously planned and executed the distribution of 30 questionnaires to the pre‐selected panel of experts. These experts, who were chosen based on stringent inclusion criteria from diverse fields relevant to biosafety and nursing, were contacted via email, accompanied by detailed instructions and explanations regarding the significance and purpose of the questionnaire. The questionnaire was designed to solicit their insights and judgements on the index system of the biosafety incident response competence for nursing staff. We received 28 effective questionnaires in return, yielding an impressive effective recovery rate of 93.33%.

In the second round, 28 questionnaires were distributed and 28 effective questionnaires were returned, with an effective recovery rate of 100%. There were respectively 24 and 5 experts putting forward specific suggestions on the index system of the biosafety incident response competence for nursing staff, which indicated the enthusiasm of the experts was relatively high (Table 2). These suggestions spanned a wide range of aspects, from the granular details of individual indicators to the overarching structure and weighting of different levels of indicators.

**TABLE 2:** The effective recovery rates of the two rounds of experts.

Type	First round			Second round		
	Number of distribution	Number (rate) of recovery	Number (rate) of proposal	Number of distribution	Number (rate) of recovery	Number (rate) of proposal
Experts in the three prevention research fields	6	5 (83.33%)	4 (66.67%)	5	5 (100.00%)	1 (20.00%)
Epidemiologists	9	8 (88.89%)	6 (66.67%)	8	8 (100.00%)	2 (25.00%)
Experts from the military health service	7	7 (100.00%)	7 (100.00%)	7	7 (100.00%)	1 (14.29%)
Clinical medical and nursing experts	8	8 (100.00%)	7 (87.50%)	8	8 (100.00%)	1 (12.50%)
Total	30	28 (93.33%)	24 (80.00%)	28	28 (100.00%)	5 (17.86%)

### 3.2 Expert Authority Coefficient

Expert authority coefficient measures the authority of the expert consulted by the letter, which is usually expressed by the expert judgement basis and expert familiarity, and is the average of the two, and the expert authority coefficient greater than 0.7 indicates that the letter inquiry result is reliable (Heed et al. 2022). The basis of expert judgement is mainly evaluated from four aspects: practical experience, theoretical analysis, reference to domestic and foreign data, and intuitive feeling. Expert familiarity is rated on a 5‐point scale, from ‘*very familiar*’ to ‘*unfamiliar*’ with a score of ‘0.9’ to ‘0.1’. In this study, the coefficient of expert judgement by letter consultation was 0.911 (Table 3), the coefficient of expert familiarity was 0.843 (Table 4) and the coefficient of expert authority was 0.877, which met the standard that the coefficient of expert authority by letter consultation should be greater than 0.7, indicating a high degree of expert authority.

**TABLE 3:** The coefficient of expert judgement by letter consultation (Ca) frequency.

Basis of judgement	High		Medium		Low	
	Times	Frequency (%)	Times	Frequency (%)	Times	Frequency (%)
Theoretical analysis	19	67.86	9	32.14	0	0.00
Practical experience	12	42.86	16	57.14	0	0.00
Reference	17	60.71	11	39.29	0	0.00
Intuitive feeling	6	21.43	8	28.57	14	50.00

**TABLE 4:** The coefficient of expert familiarity (Cs) frequency.

Familiarity	Very familiar		Quiet familiar		Generally familiar		Not so familiar		Unfamiliar	
	Times	Frequency (%)	Times	Frequency (%)	Times	Frequency (%)	Times	Frequency (%)	Times	Frequency (%)
Self‐assessment	20	71.43	8	28.57	0	0.00	0	0.00	0	0.00

### 3.3 Expert Opinions' Coordination Degree

The degree of coordination of expert opinions is used to judge the consistency of the expert's recognition of the item, usually expressed by the Kendall harmony coefficient (Kendall's W). Kendall's W results range between 0 and 1, and a value closer to 1 indicates that the degree of expert coordination is better (Orvieto et al. 2021). In the first round, the Kendall's concordance coefficients of the first‐, second‐ and third‐level indicators were 0.372, 0.384 and 0.301, respectively. These initial values, while not extremely high, were a reflection of the natural diversity of perspectives that emerged when experts from different backgrounds and with varying experiences first engaged with the complex topic of biosafety incident response competence for nursing staff. However, as the consultation progressed to the second round, after the experts had the opportunity to review and reflect on each other's comments from the first round, the Kendall's concordance coefficients of the first‐, second‐ and third‐level indicators improved to 0.423, 0.401 and 0.424, respectively. This upward trend signalled a growing convergence of ideas and a strengthening of consensus. The Kendall's W test had statistical significance (*p* < 0.01), which means that the experts are in agreement (Table 5).

**TABLE 5:** The result of expert opinions' coordination degree.

Round	Index level	Kendall's W	χ^2^	*p*
Round 1	First level	0.372	31.216	< 0.01
	Second level	0.384	96.732	< 0.01
	Third level	0.301	395.716	< 0.01
Round 2	First level	0.423	35.542	< 0.01
	Second level	0.401	101.103	< 0.01
	Third level	0.424	569.201	< 0.01

### 3.4 Expert Opinions' Concentration Degree

The degree of concentration of expert opinions was reflected by the average of items and the coefficient of variation, which reflected the degree of dispersion on the unit mean and was expressed by the ratio of standard deviation to the mean. The higher the average score of experts on the item, the greater the importance of the item; the smaller the coefficient of variation of the entry, the more concentrated the expert opinion. If the average score of the item importance assignment was < 3.5 points or the coefficient of variation was > 0.25, the index item is deleted (Zhang et al. 2022).

In the results of the first round of our correspondence study, two specific entries, namely, ‘understand the main points of epidemic prevention work and regularly carry out regular epidemic prevention and control work inside and outside the hospital’ and ‘can correctly mark and delineate biosafety contaminated areas’, fell short of the threshold. Their importance scores were < 3.5, and the coefficient of variation was > 0.25. Upon in‐depth discussion among the research team and with the input of the experts, it was determined that these items had several issues. For the former, although epidemic prevention work was undeniably crucial, the way it was phrased was too broad and lacked specificity in the context of nursing staff's direct role in biosafety incident response. It encompassed activities that were perhaps better addressed in broader public health frameworks rather than being a focused indicator for nursing competence. The latter item, ‘can correctly mark and delineate biosafety contaminated areas’, had a high degree of variability in expert opinions due to differences in regional practices, hospital protocols and the level of detail expected in such markings. After careful consideration, these two items were deleted to streamline the index system and enhance its precision. Thankfully, in the second round of correspondence, no items met the deletion criteria, signifying a more stable and agreed‐upon set of indicators had emerged.

### 3.5 Expert Opinions’ Sorting

The first round of our Delphi survey was a hive of activity, with extensive modifications, mergers, adjustments, additions and deletions of entries, to optimise the index system. Thirteen entries were modified, with these modifications ranging from subtle rewording to significant restructuring of the content to better align with the overall goals of assessing nursing staff's biosafety incident response ability. Five entries were merged, often when two or more items were found to be overlapping in concept or function, and combining them into a single, more comprehensive indicator enhanced the clarity and simplicity of the system. Three entries were adjusted in terms of their scope, weighting or placement within the hierarchical structure, ensuring a more logical flow and balance. Six entries were added, filling gaps in the knowledge, skills and attitude domains that had been identified during the initial review and expert consultations. These new entries focused on emerging trends in biosafety, such as new disinfection techniques, updated personal protective equipment (PPE) handling procedures and enhanced communication strategies during incidents. Lastly, two entries were deleted, as per the concentration degree analysis discussed earlier.

**FIGURE 1:**
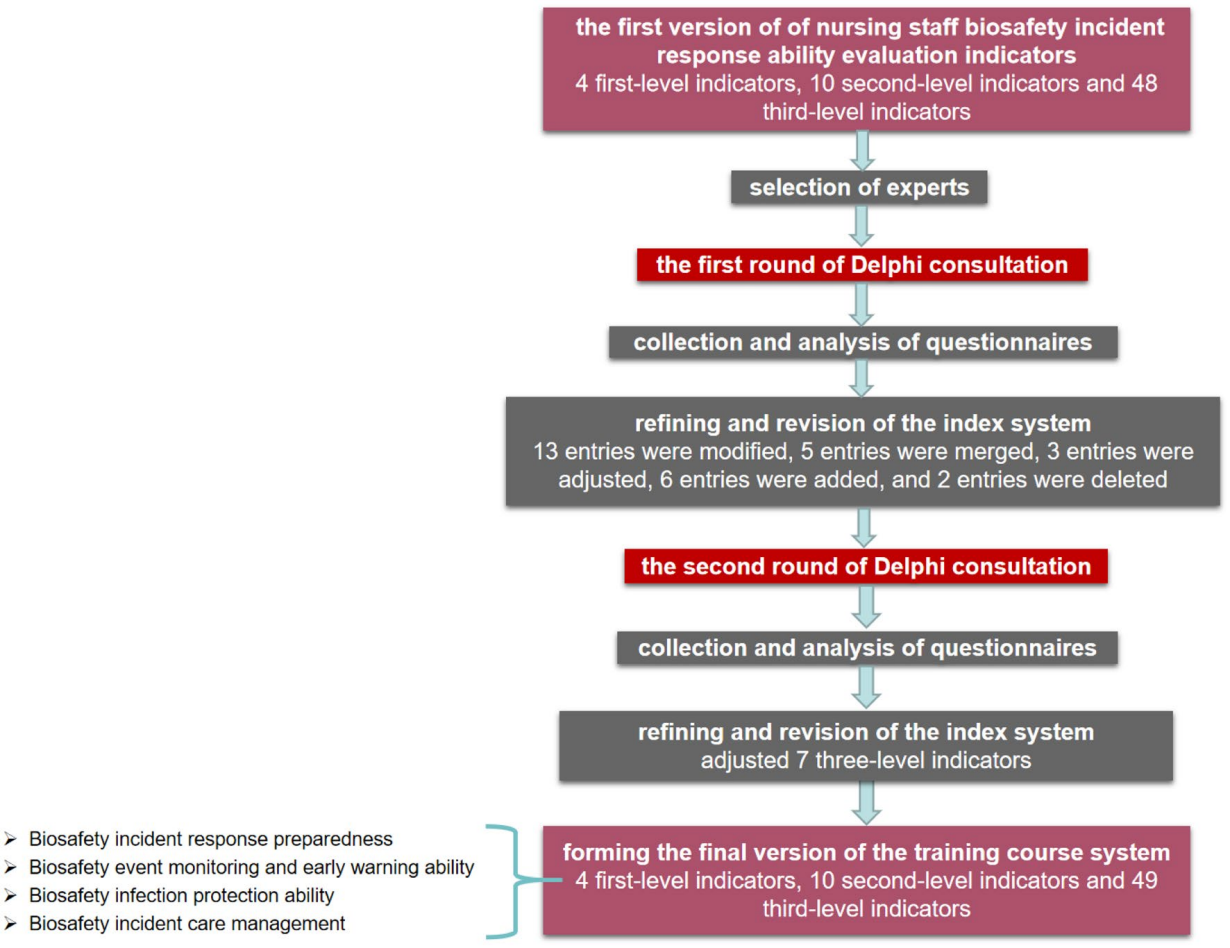
The entire process of the Delphi study.

In the second round, building on the progress made in the first round, we fine‐tuned the index system further by adjusting 7 three‐level indicators. These adjustments were made with the goal of achieving greater coherence and comprehensiveness within the overall structure. Through this iterative process, we finally arrived at a well‐defined set of evaluation indicators, comprising 4 first‐level indicators that provided a broad framework for the assessment, 10 second‐level indicators that drilled down into more specific aspects of biosafety incident response and 49 third‐level indicators that offered granular details and actionable criteria for evaluating nursing staff's biosafety incident response ability (Figure 1, Table 6).

**TABLE 6:** The index system of the biosafety incident response competence for nursing staff.

Index level 1st, 2nd and 3rd	Min	Max	Mean	SD	Variable coefficient
1 Biosafety incident response preparedness	3.00	5.00	4.250	0.518	0.122
1.1 Theoretical knowledge	4.00	5.00	4.500	0.509	0.113
1.1.1 Understand biosafety definitions, categories, hazards and current or future potential national and international biosafety risks	3.00	5.00	4.679	0.612	0.131
1.1.2 Understand relevant laws and regulations such as the Biosafety Law of the People's Republic of China, the Law of the People's Republic of China on the Prevention and Control of Infectious Diseases, and the Regulations on Biosafety Management of Pathogenic Microorganism Laboratories	3.00	5.00	4.429	0.742	0.168
1.1.3 Be familiar with biosafety incidents involving paramedics that require paramedic involvement	3.00	5.00	4.607	0.629	0.137
1.1.4 Grasp the knowledge of care for common symptoms of patients with biological infections such as fever, chills, dizziness, headache, nausea, vomiting, diarrhoea, rash, dyspnea, convulsions and disturbance of consciousness	3.00	5.00	4.679	0.612	0.131
1.1.5 Understand the types of pathogenic microorganisms and the transmission routes of different types of pathogenic microorganisms	3.00	5.00	4.679	0.548	0.117
1.1.6 Be familiar with the concept of antimicrobial resistance and the use of antimicrobials	3.00	5.00	4.714	0.535	0.113
1.1.7 Understand the biosafety management and classification requirements of pathogenic microorganism laboratory	3.00	5.00	4.214	0.630	0.149
1.1.8 Understand the types of biological warfare agents, the ways of bioterrorism attacks, the characteristics of bioterrorism attacks and the characteristics of biosecurity responses in modern warfare	3.00	5.00	4.071	0.663	0.163
1.2 Practical response preparation	4.00	5.00	4.500	0.509	0.113
1.2.1 Be familiar with biosafety medical emergency command system, mobile force deployment and emergency plan	3.00	5.00	3.893	0.786	0.202
1.2.2 Be familiar with the use of biosafety emergency drugs and reserve requirements of nursing prevention and control materials	3.00	5.00	3.857	0.756	0.196
1.2.3 Regularly participate in biosafety medical rescue exercises and training, and joint military and civilian rescue exercises to deal with emergencies	3.00	5.00	4.000	0.770	0.192
1.2.4 Regularly participate in the education of biosafety‐related science knowledge	3.00	5.00	4.500	0.638	0.142
1.2.5 Regularly pay attention to the biosafety frontier and regularly participate in the training of biosafety nursing skills	4.00	5.00	4.714	0.460	0.098
2 Biosafety event monitoring and early warning ability	3.00	5.00	4.000	0.667	0.167
2.1 Biosafety risk monitoring and identification	3.00	5.00	4.143	0.705	0.170
2.1.1 Monitoring of hospital infection risk	4.00	5.00	4.679	0.476	0.102
2.1.2 Monitoring of common symptoms in patients with biological infections	4.00	5.00	4.679	0.476	0.102
2.1.3 Monitoring of microbial resistance	3.00	5.00	4.536	0.576	0.127
2.1.4 Ability to identify biosafety risks	3.00	5.00	4.536	0.576	0.127
2.2 Biosafety risk quarantine and screening	3.00	5.00	4.000	0.770	0.192
2.2.1 Understand the quarantine points and requirements of public goods, environment, medical equipment and equipment	3.00	5.00	4.179	0.772	0.185
2.2.2 Understand the main points and requirements of detection and screening of pathogenic microorganisms and drug‐resistant bacteria	3.00	5.00	4.000	0.720	0.180
2.2.3 Acquire the correct collection methods of blood culture samples and nasopharyngeal swabs from patients with biological infection	3.00	5.00	4.500	0.638	0.142
2.3 Biosafety risk assessment and reporting	3.00	5.00	3.893	0.685	0.176
2.3.1 Possess the ability to observe and evaluate the injury of patients with biological infection	4.00	5.00	4.750	0.441	0.093
2.3.2 Possess the ability to assess the harm of pathogenic microorganisms	4.00	5.00	4.786	0.418	0.087
2.3.3 Be able to comprehensively predict and evaluate the risk of potential complications in patients with biological infections	4.00	5.00	4.893	0.315	0.064
2.3.4 Possess the ability to assess biosafety incident level, radiation impact range, severity and medical rescue response level	3.00	5.00	4.357	0.731	0.168
2.3.5 Grasp the reporting requirements, reporting time limit, reporting content and reporting process of different types of biosafety incidents	3.00	5.00	4.393	0.737	0.168
3 Biosafety infection protection ability	4.00	5.00	4.429	0.504	0.114
3.1 Protection ability	4.00	5.00	4.607	0.497	0.108
3.1.1 Grasp the connotation of standard prevention and additional prevention	4.00	5.00	4.964	0.189	0.038
3.1.2 Acquire specific protection requirements and measures for different types and levels of biosafety incidents	4.00	5.00	4.857	0.356	0.073
3.1.3 Grasp the procedures and methods of putting on and taking off biosafety protective equipment such as isolation suit, protective suit and gas mask	4.00	5.00	4.929	0.262	0.053
3.1.4 Grasp the emergency treatment process of skin and mucous membrane exposure, respiratory mucous membrane injury, sharp instrument injury and other biosafety occupational exposure and injury	4.00	5.00	4.964	0.189	0.038
3.1.5 Understand the vaccination of biosafety protective vaccines	3.00	5.00	4.500	0.745	0.166
3.2 Infection control ability	4.00	5.00	4.571	0.504	0.110
3.2.1 Understand the isolation requirements for different types of biosecurity incident sites	3.00	5.00	4.429	0.690	0.156
3.2.2 Strengthen nosocomial infection control to reduce the occurrence of drug‐resistant bacterial infection	4.00	5.00	4.786	0.418	0.087
3.2.3 Be able to properly handle blood, body fluids, secretions, excreta and biosafety‐related medical waste from patients with biological infections	4.00	5.00	4.893	0.315	0.064
3.2.4 Grasp all kinds of decontamination technology	3.00	5.00	4.393	0.737	0.168
3.2.5 Grasp the methods of biological warfare agent removal	3.00	5.00	4.464	0.693	0.155
4 Biosafety incident care management	4.00	5.00	4.643	0.488	0.105
4.1 Basic care ability	4.00	5.00	4.821	0.390	0.081
4.1.1 Grasp the nursing and rescue process and nursing technology of common symptoms of patients with biological infection	4.00	5.00	4.964	0.189	0.038
4.1.2 Grasp the nursing rescue process and nursing technology for patients with major emerging infectious diseases	4.00	5.00	4.929	0.262	0.053
4.1.3 Grasp the emergency nursing technique of acute and critical patients with biological infection	4.00	5.00	4.964	0.189	0.038
4.1.4 Possess the ability to use antimicrobials rationally and control microbial resistance	3.00	5.00	4.536	0.576	0.127
4.1.5 Grasp the rescue process and nursing points of patients injured by biological warfare agents	3.00	5.00	4.393	0.685	0.156
4.1.6 Grasp the first aid and nursing technology of different types of injuries and major injuries caused by biological weapons	3.00	5.00	4.607	0.629	0.137
4.1.7 Possess the ability to properly transport and evacuate bioinfected patients	3.00	5.00	4.464	0.744	0.167
4.2 Psychological care ability	3.00	5.00	4.571	0.634	0.139
4.2.1 Possess a good ability to withstand pressure and psychological adjustment in the biosafety incident rescue	3.00	5.00	4.643	0.559	0.120
4.2.2 Possess the ability of psychological adjustment and psychological care for biologically infected patients and their families affected by infectious diseases and biological warfare agents	3.00	5.00	4.643	0.621	0.134
4.3 Care management ability	3.00	5.00	4.786	0.499	0.104
4.3.1 Possess the ability to coordinate and manage biosafety medical relief materials	3.00	5.00	4.643	0.559	0.120
4.3.2 Grasp the key points of medical record management and record of patients with biological infection	3.00	5.00	4.536	0.576	0.127
4.3.3 Possess the ability to manage the personnel involved in biosafety emergency rescue, and be able to reasonably organise, allocate, coordinate, coordinate, guide and manage biosafety nursing work	3.00	5.00	4.429	0.690	0.156
4.3.4 Possess the ability to communicate well with superiors and organisations to seek effective rescue assistance	3.00	5.00	4.464	0.576	0.129
4.3.5 Possess the ability to coordinate nursing collaboration between different departments in biosafety rescue	3.00	5.00	4.536	0.693	0.153

### 3.6 The Implement of Final Index in Nursing Education Programs

We used the final evaluation index system of clinical nursing staff's biosafety incident response competence in the biosafety education and training of nursing staff. This implementation is based on the evaluation index we have constructed as the foundation and framework. It will make our findings more practical in hospital and nursing education program.

## 4 Discussion

### 4.1 Reliability of Consultation Results

In this study, two rounds of Delphi expert consultation were conducted on the index system of biosafety incident response competence for nursing staff. Experts were selected from the fields of three prevention and control research, epidemiology and military health service science, and clinical medical and nursing experts were also included who had participated in biosafety rescue, which was representative and ensured the quality and reliability of the consultation results. The effective questionnaire recovery rates of the two rounds of expert consultation were 93.33% and 100%, indicating that the experts had a high degree of enthusiasm for the consultation; the authority coefficient of the consultation experts was 0.877, indicating that the authority of the consultation was assured and the differences in the Kendall harmony coefficients of the indicators at all levels of the two rounds of expert consultation were statistically significant (*p* < 0.01), indicating that the experts had a high degree of recognition of the indicator items and the experts' opinions.

### 4.2 The Significance of Constructing Index System of Biosafety Incident Response Competence for Nursing Staff

COVID‐19 has exposed the world to an unprecedented biosecurity threat, with serious implications for global politics, economy and even human health (Karthik et al. 2020; W. Zhou et al. 2022). Currently, the global biosecurity situation is becoming increasingly critical (Dittmer, Eason, and Juarez 2022). Globalisation has facilitated the spread of infectious diseases and biosecurity risks; the expansion of human activities has encroached on the territories of other organisms, and more and more viruses, bacteria and diseases have accelerated their cross‐border invasion (Callaway 2023); the double‐edged sword effect of biotechnology development has become increasingly prominent (Nelson et al. 2021). They all pose a great challenge to health care.

The nursing staff, who make up the majority of the medical team, has recently been instrumental in saving lives during biosafety emergencies related to the new coronavirus pneumonia outbreak (Cadge et al. 2021). The nursing staff has gained experience by treating infectious diseases (Song et al. 2021; Wu et al. 2021). However, their knowledge of biosafety is limited to infectious disease public health emergencies, the concept of biosafety events is unclear and the placement of ambulance duties is unclear. To improve the biosafety event response competence of nursing staff, it is essential to have a thorough understanding of biosafety events and threats, a clear understanding of biosafety event response competence and knowledge of biosafety‐related nursing.

### 4.3 Content Analysis of the Index System of Biosafety Incident Response Competence for Nursing Staff

#### 4.3.1 Biosecurity Incident Response Preparedness

Biosafety incident response preparedness consists of both theoretical knowledge reserves and practical response preparation. Previous studies have shown that the biosafety knowledge reserve of clinical nursing staff is not optimistic and they know little about biosafety prevention (Chen et al. 2019). Therefore, this part of the study systematically organises and classifies the biosafety knowledge related to nursing staff and summarises the requirements for practice preparedness competencies. In the theoretical knowledge module of biosafety incident response, the highest mean score was given to the entry ‘Be familiar with the concept of antimicrobial resistance and the use of antimicrobials’, indicating that experts consider knowledge about microbial resistance to be the most important aspect of biosafety knowledge. According to the international drug resistance assessment report, the current global drug resistance situation is not optimistic, and it is expected that 10 million people will die from drug‐resistant bacterial infections globally each year by 2050, with an economic loss of $100 trillion (La Rosa, Johansen, and Molin 2022; Munir et al. 2020). Nursing staff, as the main force on the front line, play an important role in monitoring the duration of medication administration as well as the dose of medication, and are involved in the management of medication throughout the process from the time of patient admission to the discharge phase. In this way, it is important to strengthen the concept of microbial resistance control among nurses to control the rising trend of antimicrobial drug resistance (Olans, Olans, and Witt 2017; van Huizen et al. 2021). The highest mean score in the module on practice preparation for biosafety incident response was for the item ‘Regularly pay attention to the development of biosafety frontier and regularly participate in the training of biosafety nursing skills’, indicating that experts consider nursing staff participation in training related to biosafety nursing as the most important practice preparation. The study showed that biosafety‐related training can significantly improve nursing staff's biosafety protection competence and level, and nursing staff should regularly participate in biosafety‐related training to improve biosafety competence (Bajjou et al. 2020; Kinlay et al. 2021). Basic knowledge related to this content should be integrated at the undergraduate level. This includes cultivating the ability to care for patients with common biological infection symptoms. For continuous training and postgraduate level, more in‐depth and expanded training should be provided. For example, in‐depth study of the care key points for patients with different types of biological infections such as new and sudden infectious diseases, biological warfare agents and biological weapons injuries. This hierarchical design is based on the knowledge reserve and practical needs of students at different educational stages, which helps to improve the ability of nursing staff more targeted.

#### 4.3.2 Biosafety Event Monitoring and Early Warning Abilities

Monitoring to identify biosafety threats is a necessary prerequisite for effective response to biosafety events. Nursing staff, as the front line of the clinic and in close contact with patients, need to have biosafety event monitoring and early warning abilities to identify biosafety risks, move the risk window forward, reduce the spread of hazard and block the spread of risks in time to achieve the fastest biosafety event rescue (Wang et al. 2021). The monitoring and early warning module contains three parts: biosafety risk monitoring and identification, biosafety risk quarantine and screening abilities, and biosafety risk assessment and reporting, in which the highest score is biosafety risk monitoring and identification. This entry contains monitoring of hospital infection risk, monitoring of common symptoms in patients with biological infections, monitoring of microbial resistance and the ability to identify biosafety risks. Because most biologic infections are infectious, nursing staff also need to master the proper collection of specimens, such as blood culture specimens and nasopharyngeal swabs from biologically infected patients, to achieve rapid and accurate screening of biologically infected patients (Agarwal et al. 2021). Nursing staff, as the main force in healthcare, still need to be knowledgeable about quarantine to ensure that they can quickly transform themselves as a back‐up supplement to quarantine screening in the event of a sudden large‐scale biosafety event. Nursing staff also need to have the ability to assess and report biosecurity risks, and be able to make timely reports once a biosecurity event is detected, and what is more, they are required to assess the severity level, impact and magnitude of the hazard of a biosecurity event.

#### 4.3.3 Biosafety Infection Protection Abilities

In biosafety incident response, infection protection abilities were a prerequisite for nursing staff to ensure their own safety and be able to carry out their nursing work (He et al. 2022). The pathogenic microorganisms of most biological infections are infectious and can spread rapidly in a short period of time, resulting in widespread infection of personnel (Ho, Enriquez, and Multani 2020). As a front‐line clinical force, nursing staff are the ones who have the closest contact with patients, facing the huge biosafety threat. The risk of occupational exposure of nursing staff is greatly increased (Drozd et al. 2021). Good occupational protection can effectively control occupational risk factors and effectively avoid nursing occupational risks (Alsabaani et al. 2022). A study on infection prevention and control measures for clinical nursing staff showed that the implementation of infection prevention and control measures for clinical nursing staff was not satisfactory, especially in the prevention and control of multi‐drug resistant bacteria (Kim and Hwang 2020). Nursing staff should clarify the concept of standard and additional prevention and remain biosafety vigilant at all times. The ability to properly handle blood, body fluids, secretions, excretions and biosafety‐related medical waste from biologically infected patients is the most important aspect in terms of infection control abilities, which will avoid secondary transmission or infection. Strengthening nosocomial infection control and reducing the occurrence of drug‐resistant bacterial infections is another important aspect of infection control ability, which is related to the pessimistic situation of drug‐resistant bacterial infections in the nosocomial setting (Suganya et al. 2022). Nursing staff need to have knowledge related not only to anti‐drug‐resistant bacteria but also to implement actions to strictly control the time and dosage of medication and closely observe them to effectively reduce the occurrence of drug‐resistant situations (Xu et al. 2017).

#### 4.3.4 Biosafety Incident Care Management Abilities

The biosafety incident care management ability was the highest scoring and most important one among the biosafety incident response abilities of nursing staff. Development of soft or transversal skills can maximise the safety of life of bio infected patients, reduce economic losses and maintain social stability. The biosafety event care management ability contained three parts: basic care ability, psychological care ability and care management ability, among which basic care ability weights the greatest. Good basic nursing ability was a prerequisite for nursing staff to implement life‐saving treatment for patients (Su et al. 2022). Nursing staff not only need to have the ability to care for patients with common symptoms of biological infections and patients with acute and critical biological infections but also need to understand and master the key points of care for patients with different categories of biological infections such as new and sudden infectious diseases, biological warfare agents and biological weapons injuries. Psychological care ability includes the nursing staff's own ability to resist stress, psychological adjustment and psychological care for biologically infected patients and their families. Studies have shown that nearly half of the nurses suffered from psychological distress and emotional exhaustion during the rescue of the COVID‐19, which in turn led to low productivity and increased nursing errors (Cortés‐Álvarez and Vuelvas‐Olmos 2022). Therefore, the ability of nursing staff to cope with biosafety events is not only at the level of knowledge and skills, but should also focus on their psychological tolerance and psychological health during the response to the event, and should also pay active attention to the psychological health of patients and their families. As clinical nurses, in addition to professional skills and psychological care ability in the process of biosafety event care disposal, they should also have certain nursing management ability to coordinate all kinds of matters and ensure the smooth development of nursing rescue work.

### 4.4 The Index System of Biosafety Incident Response Competence for Nursing Staff Aligning With NOP

The Nursing Outcome Prioritisation (NOP) plays a pivotal role in shaping the standards and expectations of modern nursing care. In light of this, the evaluation index system of clinical nursing staff's biosafety incident response competence that our study has meticulously developed holds significant implications when examined through the lens of NOP.

At the highest level of our index hierarchy, the 4 first‐level indicators form the backbone of the entire framework, each bearing a direct connection to the core tenets of NOP. Take, for instance, the dimension dedicated to nursing staff's professional knowledge reserve. In the context of NOP, which is resolutely committed to elevating nursing quality and fortifying patient safety, this aspect is of utmost importance. Nurses armed with a rich reservoir of biosafety knowledge are better equipped to navigate the complex web of daily nursing tasks. When faced with the exigencies of a biosafety incident, the value of this professional knowledge becomes even more pronounced. In the chaos and urgency that often accompanies such events, nurses with a comprehensive understanding of biosafety protocols, pathogen transmission mechanisms and emergency response procedures can act swiftly and decisively. They know exactly which PPE to don, how to isolate contaminated areas effectively and which communication channels to activate within the hospital's emergency response framework. By doing so, they not only safeguard themselves from potential harm but, more importantly, they minimise the risk of spreading infections among patients and healthcare workers alike, thus directly fulfilling NOP's objective of ensuring a safe healthcare environment.

Delving deeper into the index system, the 10 second‐level indicators offer a more granular perspective on nursing capabilities and further strengthen the alignment with NOP. These indicators cover a diverse range of areas such as the proper donning, doffing and maintenance of PPE. Nurses who are proficient in handling PPE, as dictated by our second‐level indicators, can significantly reduce the risk of cross‐contamination during patient care, especially in high‐risk situations like dealing with patients with contagious diseases or in the midst of a biosafety incident.

The 49 third‐level indicators, with their painstaking detail, translate the overarching goals of the first‐ and second‐level indicators into actionable steps. For example, a specific third‐level indicator might detail the exact sequence of steps for disinfecting a contaminated surface after a biosafety incident. This level of precision ensures that nurses across different healthcare settings can perform their duties in a standardised and consistent manner, which is vital for achieving the uniformity and excellence in care that NOP aspires to.

## 5 Conclusion

The index system of biosafety incident response ability of nursing staff established in this study was detailed and comprehensive, and the construction method was scientific and rigorous. By clarifying the specific items of biosafety incident response ability, it was helpful to improve the response ability of nursing staff to biosafety incidents and better cope with biosafety threats. In future research, we plan to provide training programs based on the index system, including theoretical knowledge teaching about biosafety incidents and practical skill training for response operations. Also, we will develop assessment tools related to the index to regularly evaluate the performance of nursing staff.

## 6 Limitation

Because of limited research funding and time, we sent the questionnaire to 28 experts from different regions of China. The expert panel of other health disciplines may constitute a limitation. Perspectives and knowledge of nurse's roles, competencies and responsibilities differ and can be a limitation to the real and needed nursing competencies in this field.

## Author Contributions

Chao Wu and Mengyi Hu acontributed to the research design and paper writing; Xiaolan Guo and Hongjuan Lang contributed to the distribution and collection of the questionnaire; Lu Li, Qiang Xu and Mimi Fu contributed to the research design and the data analysis and Xinyan Zhang contributed to the proofreading and English version of the paper.

## Consent

The authors have nothing to report.

## Conflicts of Interest

The authors declare no conflicts of interest.

## Supporting information


Data S1.

## Data Availability

The datasets used and analysed during the current study are available from the corresponding author on reasonable request (906963251@qq.com).
