# Supplementary material for: Construction of an Index System of the Biosafety Incident Response Capability for Nursing Staff: A Delphi Study
Source: Nurs Open. 2024 Dec 30;12(1):e70118. doi: 10.1002/nop2.70118 (PMC11684915; doi:10.1002/nop2.70118)
Supplement: Supplementary file 1 — Data S1. [file NOP2-12-e70118-s001.docx]

**Consultation questionnaire**

**(a) General information of the experts**

| Age(years) |  |
| --- | --- |
| Working years（years） |  |
| Research field |  |
| Title |  |
| Highest degree |  |
| Region |  |

**(b) The index system of the biosafety incident response capability for nursing staff consultation form**

| Index level 1^st^, 2^nd^ and 3^rd^ | Agree | Disagree | Score | Revise |
| --- | --- | --- | --- | --- |
| 1 biosafety incident response preparedness |  |  |  |  |
| 1.1 Theoretical knowledge |  |  |  |  |
| 1.1.1 Understand biosafety definitions, categories, hazards, and current or future potential national and international biosafety risks |  |  |  |  |
| 1.1.2 Understand relevant laws and regulations such as the Biosafety Law of the People's Republic of China, the Law of the People's Republic of China on the Prevention and Control of Infectious Diseases, and the Regulations on Biosafety Management of Pathogenic Microorganism Laboratories |  |  |  |  |
| 1.1.3 Be familiar with biosafety incidents involving paramedics that require paramedic involvement |  |  |  |  |
| 1.1.4 Grasp the knowledge of care for common symptoms of patients with biological infections such as fever, chills, dizziness, headache, nausea, vomiting, diarrhea, rash, dyspnea, convulsions, and disturbance of consciousness |  |  |  |  |
| 1.1.5 Understand the types of pathogenic microorganisms and the transmission routes of different types of pathogenic microorganisms |  |  |  |  |
| 1.1.6 Be familiar with the concept of antimicrobial resistance and the use of antimicrobials |  |  |  |  |
| 1.1.7 Understand the biosafety management and classification requirements of pathogenic microorganism laboratory |  |  |  |  |
| 1.1.8 Understand the types of biological warfare agents, the ways of bioterrorism attacks, the characteristics of bioterrorism attacks, and the characteristics of biosecurity responses in modern warfare |  |  |  |  |
| 1.2 Practical response preparation |  |  |  |  |
| 1.2.1 Be familiar with biosafety medical emergency command system, mobile force deployment and emergency plan |  |  |  |  |
| 1.2.2 Be familiar with the use of biosafety emergency drugs and reserve requirements of nursing prevention and control materials |  |  |  |  |
| 1.2.3 Regularly participate in biosafety medical rescue exercises and training and joint military and civilian rescue exercises to deal with emergencies |  |  |  |  |
| 1.2.4 Regularly participate in the education of biosafety-related science knowledge |  |  |  |  |
| 1.2.5 Regularly pay attention to the biosafety frontier, and regularly participate in the training of biosafety nursing skills |  |  |  |  |
| 2 Biosafety event monitoring and early warning capability |  |  |  |  |
| 2.1 Biosafety risk monitoring and identification |  |  |  |  |
| 2.1.1 Monitoring of hospital infection risk |  |  |  |  |
| 2.1.2 Monitoring of common symptoms in patients with biological infections |  |  |  |  |
| 2.1.3 Monitoring of microbial resistance |  |  |  |  |
| 2.1.4 Ability to identify biosafety risks |  |  |  |  |
| 2.2 Biosafety risk quarantine and screening |  |  |  |  |
| 2.2.1 Understand the quarantine points and requirements of public goods, environment, medical equipment and equipment |  |  |  |  |
| 2.2.2 Understand the main points and requirements of detection and screening of pathogenic microorganisms and drug-resistant bacteria |  |  |  |  |
| 2.2.3 Master the correct collection methods of blood culture samples and nasopharyngeal swabs from patients with biological infection |  |  |  |  |
| 2.3 Biosafety risk assessment and reporting |  |  |  |  |
| 2.3.1 Possess the ability to observe and evaluate the injury of patients with biological infection |  |  |  |  |
| 2.3.2 Possess the ability to assess the harm of pathogenic microorganisms |  |  |  |  |
| 2.3.3 Be able to comprehensively predict and evaluate the risk of potential complications in patients with biological infections |  |  |  |  |
| 2.3.4 Possess the ability to assess biosafety incident level, radiation impact range, severity, and medical rescue response level |  |  |  |  |
| 2.3.5 Master the reporting requirements, reporting time limit, reporting content and reporting process of different types of biosafety incidents |  |  |  |  |
| 3 Biosafety infection protection ability |  |  |  |  |
| 3.1 Protection ability |  |  |  |  |
| 3.1.1 Master the connotation of standard prevention and additional prevention |  |  |  |  |
| 3.1.2 Master specific protection requirements and measures for different types and levels of biosafety incidents |  |  |  |  |
| 3.1.3 Master the procedures and methods of putting on and taking off biosafety protective equipment such as isolation suit, protective suit and gas mask |  |  |  |  |
| 3.1.4 Master the emergency treatment process of skin and mucous membrane exposure, respiratory mucous membrane injury, sharp instrument injury and other biosafety occupational exposure and injury |  |  |  |  |
| 3.1.5 Understand the vaccination of biosafety protective vaccines |  |  |  |  |
| 3.2 Infection control capability |  |  |  |  |
| 3.2.1 Understand the isolation requirements for different types of biosecurity incident sites |  |  |  |  |
| 3.2.2 Strengthen nosocomial infection control to reduce the occurrence of drug-resistant bacterial infection |  |  |  |  |
| 3.2.3 Be able to properly handle blood, body fluids, secretions, excreta and biosafety-related medical waste from patients with biological infections |  |  |  |  |
| 3.2.4 Master all kinds of decontamination technology |  |  |  |  |
| 3.2.5 Master the methods of biological warfare agent removal |  |  |  |  |
| 4 Biosafety incident care management |  |  |  |  |
| 4.1 Basic care capacity |  |  |  |  |
| 4.1.1 Master the nursing and rescue process and nursing technology of common symptoms of patients with biological infection |  |  |  |  |
| 4.1.2 Master the nursing rescue process and nursing technology for patients with major emerging infectious diseases |  |  |  |  |
| 4.1.3 Master the emergency nursing technique of acute and critical patients with biological infection |  |  |  |  |
| 4.1.4 Possess the ability to use antimicrobials rationally and control microbial resistance |  |  |  |  |
| 4.1.5 Master the rescue process and nursing points of patients injured by biological warfare agents |  |  |  |  |
| 4.1.6 Master the first aid and nursing technology of different types of injuries and major injuries caused by biological weapons |  |  |  |  |
| 4.1.7 Possess the ability to properly transport and evacuate bioinfected patients |  |  |  |  |
| 4.2 Psychological care ability |  |  |  |  |
| 4.2.1 Possess a good ability to withstand pressure and psychological adjustment in the biosafety incident rescue |  |  |  |  |
| 4.2.2 Possess the ability of psychological adjustment and psychological care for biological infected patients and their families affected by infectious diseases and biological warfare agents |  |  |  |  |
| 4.3 Care management ability |  |  |  |  |
| 4.3.1 Possess the ability to coordinate and manage biosafety medical relief materials |  |  |  |  |
| 4.3.2 Master the key points of medical record management and record of patients with biological infection |  |  |  |  |
| 4.3.3 Possess the ability to manage the personnel involved in biosafety emergency rescue, and be able to reasonably organize, allocate, coordinate, coordinate, guide and manage biosafety nursing work |  |  |  |  |
| 4.3.4 Possess the ability to communicate well with superiors and organizations to seek effective rescue assistance |  |  |  |  |
| 4.3.5 Possess the ability to coordinate nursing collaboration between different departments in biosafety rescue |  |  |  |  |

**(c) Expert familiarity with the content of the survey and index judgement**

| Basis of judgement | High | Medium | Low |
| --- | --- | --- | --- |
| Theoretical analysis |  |  |  |
| Practical experience |  |  |  |
| Reference |  |  |  |
| Intuitive feeling |  |  |  |

| Familiarity | Very familiar | Quiet familiar | Generally familiar | Not so familiar | Unfamiliar |
| --- | --- | --- | --- | --- | --- |
| Self-assessment |  |  |  |  |  |
